# Effects of Neuronic Shutter Observed in the EEG Alpha Rhythm

**DOI:** 10.1523/ENEURO.0171-20.2020

**Published:** 2020-10-06

**Authors:** Kevin E. Alexander, Justin R. Estepp, Sherif M. Elbasiouny

**Affiliations:** 1 Department of Biomedical, Industrial, and Human Factors Engineering, College of Engineering and Computer Science, Wright State University, Dayton, OH 45435; 2 711th Human Performance Wing, Air Force Research Laboratory, Wright-Patterson AFB, OH 45433; 3 Department of Neuroscience, Cell Biology, and Physiology, Boonshoft School of Medicine and College of Science and Mathematics, Wright State University, Dayton, OH 45435; 4 Oak Ridge Institute for Science and Education, Air Force Research Laboratory, Wright-Patterson AFB, OH 45433

**Keywords:** alpha rhythm, EEG, visual-evoked potential, neuronic shutter, visual conduction delay

## Abstract

The posterior alpha (α) rhythm, seen in human electroencephalogram (EEG), is posited to originate from cycling inhibitory/excitatory states of visual relay cells in the thalamus. These cycling states are thought to lead to oscillating visual sensitivity levels termed the “neuronic shutter effect.” If true, perceptual performance should be predictable by observed α phase (of cycling inhibitory/excitatory states) relative to the timeline of afferentiation onto the visual cortex. Here, we tested this hypothesis by presenting contrast changes at near perceptual threshold intensity through closed eyelids to 20 participants (balanced for gender) during times of spontaneous α oscillations. To more accurately and rigorously test the shutter hypothesis than ever before, α rhythm phase and amplitude were calculated relative to each individual’s retina-to-primary visual cortex (V1) conduction delay, estimated from the individual’s C1 visual-evoked potential (VEP) latency. Our results show that stimulus observation rates (ORs) are greater at a trough than a peak of the posterior α rhythm when phase is measured at the individual’s conduction delay relative to stimulus onset. Specifically, the optimal phase for stimulus observation was found to be 272.41°, where ORs are 20.96% greater than the opposing phase of 92.41°. The perception-phase relationship is modulated by α rhythm amplitude and is not observed at lower amplitude oscillations. Collectively, these results provide support to the “neuronic shutter” hypothesis and demonstrate a phase and timing relationship consistent with the theory that cycling excitability in the thalamic relay cells underly posterior α oscillations.

## Significance Statement

After accounting for neural conduction delays, we found that threshold intensity stimuli are observed at higher rates when the α wave is at a trough phase than at a peak phase, but only when α amplitude is high. Our results were derived using methods consistent with a specific hypothesis about a mechanism of visual perception, considering the structure, physiology, and transmission delays in the visual system. The results of this rigorous study design add support to the neuronic shutter hypothesis and are consistent with the theory that posterior α reflects cycling excitability in thalamic relay cells, thereby gating the flow of visual information.

## Introduction

The posterior alpha (α) rhythm, a 7- to 13-Hz oscillation, is a notable characteristic of the human electroencephalogram (EEG) over the occipital cortex, especially when eyes are closed (Berry and Wagner, 2015). This is thought to assist attention regulation, as its characteristics are often found to correlate with attention over specific sensory cortices (Sauseng et al., 2005; Ben‐Simon et al., 2013), possibly a gating mechanism to limit the flow of sensory information (Cooper et al., 2003; Janssens et al., 2018). This rhythm has analogs in the sensorimotor (Pineda, 2005; Bazanova and Vernon, 2014) and auditory cortices (Tiihonen et al., 1991; Weisz et al., 2011), with both appearing to provide similar gating. This regulatory function is further demonstrated by the observation that α activity increases during introspective behavior (Ancoli and Green, 1977) and behaviors involving internally directed attention such as imagination (Cooper et al., 2003), mental arithmetic (Ray and Cole, 1985), autobiographical recall (Yue et al., 2013), and meditative practices (Tenke et al., 2017), presumably because external sensory information is distractive during these behaviors.

However, the cellular mechanism and functional significance of α oscillations remains unclear. In the 1950s, α oscillations were proposed to represent a “neuronic shutter” (Callaway and Alexander, 1960) to down-sample incoming sensory information to reduce processing load, and similar discrete perceptual processes are proposed today (VanRullen and Koch, 2003; VanRullen et al., 2014). This shutter is thought to occur at the lateral geniculate nucleus (LGN), which relays visual information to primary visual cortex (V1). At times, LGN relay cells burst-fire at 10 Hz, with a hyperpolarized refractory period between bursts (Lopes da Silva, 1991; Sherman, 2001; Timofeev and Bazhenov, 2005; Alexander et al., 2006; Timofeev and Chauvette, 2011). If many of these cells fire synchronously, visual afferentation during a widespread refractory period is less likely to be relayed to V1, resulting in a 10-Hz visual shutter. Each burst in LGN relay cells results in a large EPSP at V1, measured as a negativity in the occipital EEG (Timofeev and Bazhenov, 2005), with higher amplitudes indicating more synchronous excitation (Pfurtscheller et al., 1996), resulting, as hypothesized by this model, from LGN excitatory volleys. Thus, the α rhythm is thought to reflect cycling excitability in the LGN visual-relay cells, which would result in cycling visual sensitivity in phase with the α oscillation. Evidence of this mechanism producing α oscillations has been found in animal models with the associated phasic gating of sensory transfer (Lorincz et al., 2009; Chen et al., 2016). The hypothesis of the present study was based on this model; however, we did not directly test the underlying cellular mechanism.

To more accurately measure the α phase relationship to perceptual performance, we accounted for neural conduction delays: if a stimulus strikes the retina at *t0*, that information arrives at the LGN at a later time, *t1*. Therefore, the *t1* LGN excitatory state determines whether the information is relayed to V1. Further, the LGN excitatory state is measurable even later in the EEG, as V1 EPSPs at *t2*. Thus, *t0* to *t2* represents the retina-to-V1 conduction delay: At *t2*, we can assess the *t1* LGN excitability, the time of afferentation of the *t0* stimulus. This is the first study, to our knowledge, to directly examine the relationship between visual observation and spontaneous α phase relative to each individual’s conduction delay, which advances the accuracy and rigor for testing the shutter hypothesis.

This study’s objective is to more robustly test the hypothesis that posterior α reflects cyclic excitability of the visual system with a phase and timing relationship predicted by underlying physiological model and conduction delays. Therefore, we presented visual stimuli (*t0*) to participants during spontaneous occipital α activity, then measured α amplitude and phase at *t2* (estimated by the individual’s C1 visual-evoked potential (VEP) component peak latency; Di Russo et al., 2002). We predicted that visual stimuli at *t0* would be more likely observed with α phase at a trough at *t2* (assumed to indicate *t1* LGN excitation) than at a peak (*t1* LGN inhibition). Additionally, this effect was predicted to be greater at high α amplitudes, indicating greater cellular excitatory state synchrony. We found that participants responded to 20.96% more stimuli with α phase at a trough versus a peak at *t2* when α amplitude was high. At low amplitude, no significant phase effect on observation rate (OR) was observed. These results support our hypothesis that the α rhythm reflects cyclic excitatory states in the visual system resulting in a visual shutter effect.

## Materials and Methods

### Participants

α Activity varies largely both within (Gonçalves et al., 2006) and between (Wieneke et al., 1980) individuals. For this study, we needed to ensure stimuli were able to be presented during times of observable (e.g., stationary) α oscillations and therefore selected participants who more readily and reliably generated observable occipital α activity. This selection was made by observing EEG activity as the participants practiced the visual sensitivity task with eyes closed during an earlier portion of the experimental session. Participants who did not readily and reliably produce occipital α oscillations during this practice period were excluded from further participation.

In total, 41 participants were recruited for this study. Based on the observable α criteria described above, 21 participants were excluded from further participation. The remaining 20 healthy participants (10 males/10 females, mean age: 22.3 years, range: 18–28 years) completed two tasks: one to estimate their individual conduction delay; the other to investigate their visual sensitivity at different phases of α oscillations. The experimental protocol was approved by the Institutional Review Boards of Wright State University and the Air Force Research Laboratory. All participants were compensated for their time.

### Recording

All recordings were made using the BioSemi ActiveTwo system (BioSemi B.V.). Recordings were made with a 2048-Hz sampling rate at 64 channel locations based on the modified combinatorial nomenclature extension of the 10–10 system (American Electroencephalographic Society, 1994) excluding the inferior chain with the exception of the channels P9/P10 and Iz (Seeck et al., 2017), with bilateral electrodes on the mastoid process, infraorbital, and outer canthus locations. Participant responses were recorded using a low-latency mechanical keyboard (Cherry MX 6.0 [G80-3930], Cherry GmbH). This was combined with other task-state and visual-stimulus timing information via light sensors placed on the monitor to record events on the ActiveTwo’s 16-bit trigger line (StimTracker 1G, Cedrus Corporation).

### Stimuli

All tasks and stimuli were constructed and presented using MATLAB (R2011b; The MathWorks) and the Psychophysics Toolbox (v3.0.13; Brainard, 1997; Pelli, 1997; Kleiner et al., 2007). This software was run on a Dell Precision T3610 computer (Dell Inc.) with the Windows 7 Professional operating system (Microsoft Corporation). Stimuli were presented to participants on a 24.5”, 240 Hz monitor (BenQ ZOWIE XL2540, BenQ Corporation) providing 4.2-ms temporal resolution for stimulus presentation. Where relevant, stimulus luminance was measured using a light meter (Light Meter LUX/FC 840020, SPER SCIENTIFIC). The participant’s head position was fixed using a chinrest placed 58 cm from this monitor. The experiment was conducted in a dark room with a natural sound machine (Dohm Classic, Marpac LLC) to mask noise disturbances.

### Data analysis

All data analyses were performed in MATLAB (R2019b; The MathWorks) using the EEGLAB Toolbox (v2019.0; Delorme and Makeig, 2004) and the ERPLAB plugin (v7.0.0; Lopez-Calderon and Luck, 2014). Statistical analyses were conducted using MATLAB’s Statistics and Machine Learning Toolbox (R2019b; The MathWorks). This software was run on a Lenovo ThinkPad P50 computer (Lenovo) with the Windows 10 Enterprise operating system (Microsoft Corporation).

### Code accessibility

Task design and data analysis code are made publicly available at https://osf.io/qnryf/; DOI 10.17 605/OSF.IO/QNRYF in addition to the recorded EEG data.

### Estimation of t2

**Figure 1.**
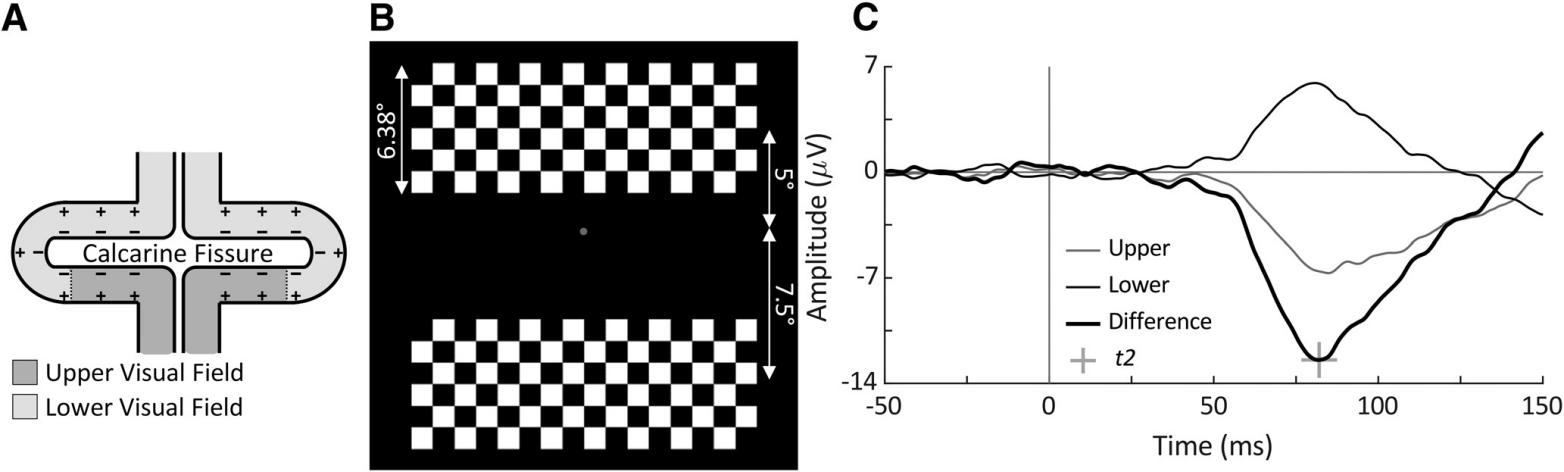
***A***, According to the cruciform model of the calcarine fissure in the V1 cortical area, the lower portion of the fissure will respond to upper visual field stimuli and vice versa for the upper portion of the fissure. For this reason, and as the upper and lower portions of the fissure contain opposing dipoles, upper and lower visual field stimuli will create waveforms of opposing polarity in the EEG. ***B***, Upper and lower visual field stimuli used for C1 VEP task are centered 3° below the fixation point to account for the overrepresentation of the lower visual field in the calcerine fissure as shown in ***A***. ***C***, C1 VEP from a single participant in response to the upper and lower stimuli, the difference was measured as the lower minus the upper VEP. The peak latency of the difference wave (indicated as +) was used as the estimate of *t2*.

The peak latency of each participant’s C1 VEP component was used as an estimate of their individual retina-to-V1 conduction delay, *t2*. This component reflects the arrival of a volley of visual information to V1 from the LGN along the optic radiations. This is analogous to the α wave, in the sense that it also reflects excitatory burst volleys from the LGN to V1, according to the hypothesized model. The C1 peak, rather than onset, latency was used to estimate the middle point of these volleys rather than their onset. Because of the specific folding of the V1 cortical area around the calcarine fissure (Fig. 1*A*), the C1 wave shape will vary with the location of the stimulus in the visual field. Stimuli presented horizontally centered in the visual field will stimulate spatially opposing V1 neurons whose dipoles will cancel each other out, yielding no measurable C1 component in the EEG (Clark et al., 1994; Di Russo et al., 2002). This is similarly the case for stimuli presented ∼3° below the vertical center (Clark et al., 1994; Di Russo et al., 2002). However, with some horizontal spacing, bilateral stimuli presented above −3° in the visual field will produce a negative C1 wave, while those presented below −3° in the visual field will produce a positive C1 wave as shown in Figure 1*B* (Clark et al., 1994; Di Russo et al., 2002).

C1 waves are found to be measured maximally at electrode POz using an averaged mastoid reference, but they are still quite small in amplitude. To obtain a well-defined C1 VEP component, both negative and positive components were obtained using upper and lower visual field stimuli. Next, the difference between these two signals was taken to create a large-amplitude wave with a clear peak (Fig. 1*C* shows these waveforms from a single participant). This peak latency was taken as an estimate of *t*2.

#### Experimental design

The stimulus used for this task was a 17° wide, 6.38° tall black and white checkerboard made of 16 × 6 1.06° squares placed on an otherwise black screen with a fixation point placed in the center (Fig. 1*B*). The checkerboard was flashed with its center either 5° above or 7.5° below the fixation point and centered horizontally. The size and placement of these stimuli were selected to generate maximum amplitude waves (Clark et al., 1994). A total of 600 upper and 600 lower stimuli were presented in a mixed random order each with a duration of 33 ms with random 250- to 450-ms interstimulus intervals, resulting in a trial time of ∼7.5 min.

Similarly to Di Russo et al. (2002), participant attention and gaze were maintained on the fixation point by a sham task performed during the trial. The fixation point would occasionally flash to a brighter color for 12.6 ms, and the participant was instructed to watch for this flash and to respond by pressing the space bar.

#### Analysis

For the C1 VEP analysis, the originally recorded 2048-Hz sampling rate was maintained but re-referenced to averaged mastoids. All EEG signals were bandpass filtered from 0.01 to 50 Hz at −6 dB, using a second order IIR Butterworth filter as implemented in the ERPLAB toolbox (Lopez-Calderon and Luck, 2014), to achieve a zero-phase filter response. A bipolar vertical electrooculogram (vEOG) signal was created by subtracting the averaged left and right infraorbital electrodes from the averaged Fp1 and Fp2 electrodes.

The data were epoched from 50 ms before to 200 ms after stimulus presentation for both the upper and lower visual stimuli. Blinks within these epochs were detected by sliding a 150-ms window at 75-ms steps over the vEOG signal, and any window containing a peak-to-peak amplitude of 100 μV or greater was rejected from analysis. Separate average VEPs were calculated for the upper and lower stimulus trials and were baseline-corrected to the 50-ms prestimulus period. The lower visual field VEP was then subtracted from the upper field VEP to create a difference wave containing a C1 wave with a clear peak.

The C1 peak latency was measured at electrode POz by finding the most negative peak within the 0- to 110-ms time window relative to stimulus onset. This peak latency was calculated for each participant individually and was used as an estimate of their individual *t2*.

### Measuring ORs

This task was designed to be conducted with closed eyes to evoke more frequent (Legewie et al., 1969) and higher amplitude α oscillations (Barry et al., 2007). For this purpose, stimuli were designed such that they could be observed as light flashes through closed eyelids. The brightness of these flashes was designed to be near threshold intensity (where participants reported seeing ∼50% of the stimuli) to prevent any ceiling or floor effects on performance.

#### Experimental design

**Figure 2.**
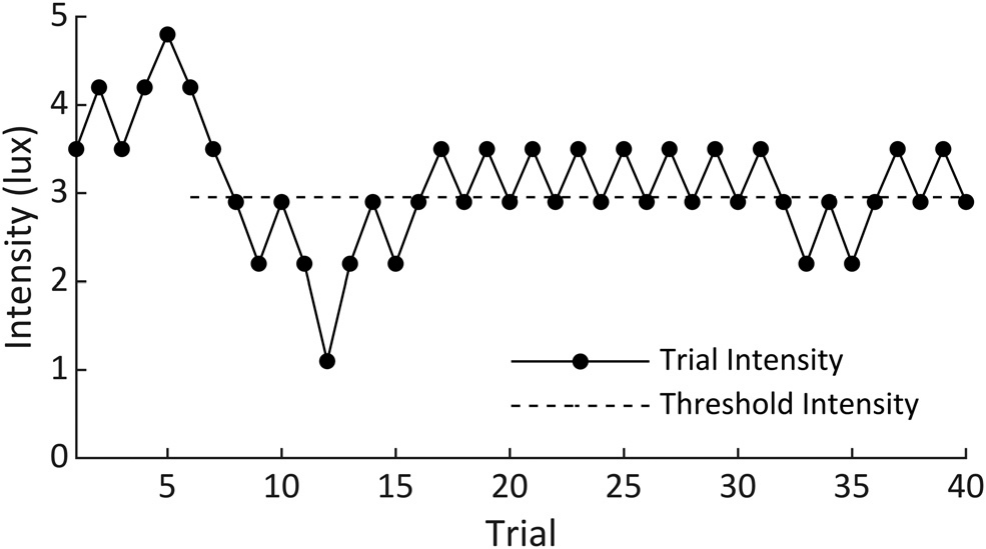
Example of the staircase method used to determine the threshold intensity in a single participant. Each time the participant observed the stimulus, the intensity in the next trial was decreased. If the participant did not observe the stimulus in one trial, the intensity was increased in the next trial. Threshold intensity was determined as the average intensity of all trials (trials 6–40 in this example) following the third reversal, or corner, in the trial-intensity trace (trial 5 in this example). The dashed threshold intensity line in this figure spans the trials over which the intensities were averaged.

The stimulus used for this task was an 8.5° square centered on an otherwise black (0.7 lux) screen. The stimulus brightness was defined by coloring the square as a greyscale value from 0 (completely black; 0.7 lux) to 255 (completely white; 89.2 lux). The stimulus was designed to be observable through the closed eyes of the participant. The brightness of the stimulus was adjusted to near the threshold intensity for each individual participant, the value at which the participant reported observing the stimulus half of the number of times it was presented. This threshold was estimated using the staircase method as described in Cornsweet (1962) during a calibration task before the main task. In this task, the participant kept their eyes closed as a series of flashes were presented. Each time the participant reported observing a flash (using the spacebar), the stimulus intensity was decreased in the next trial. However, if the participant did not report observing the stimulus, the intensity was increased on the next trial. Following the methods described in Cornsweet (1962), threshold intensity is defined as the average of all trials following the third reversal in trial-intensity slope. An example of this task from one participant is shown in Figure 2. In this figure, the third reversal occurred at trial 5, and threshold intensity was calculated as the average of trials 6–40.

The stimuli used were so dim that the participants’ ability to perceive them was sensitive to how well their eyes had adapted to the dark. Pilot testing of the task revealed that, without an adjustment period, the threshold brightness found during the calibration task would become easier to see over the course of the main task. Although visual adaptation to the dark will continue over many hours, the most rapid changes occur in the first 15 min, and visual acuity begins to plateau in 20 min (Bierings et al., 2018). Therefore, participants were given 25 min in the dark as an adaptation period before conducting the calibration task to estimate the threshold brightness.

In the main task, the stimuli were flashed for a period of 8.4 ms, and the participant was instructed to respond using the keyboard any time they observed a flash. The participant had to respond with the space bar within 2 s of the stimulus to be a valid response. The experimenter triggered the stimuli during the times of occipital α oscillation as observed in the real-time data throughout the task. After the experimenter triggered the stimulus, it was presented after a random interval of <750 ms (to mitigate potential confounding effects of experimenter bias in stimulus presentation timing).

#### Analysis

All data were down-sampled to 512 Hz and re-referenced to averaged mastoids. The signal was bandpass filtered from 0.01 to 50 Hz at –6 dB using a second order IIR Butterworth filter, as implemented in the ERPLAB toolbox (Lopez-Calderon and Luck, 2014), to achieve a zero-phase filter response. A bipolar vEOG signal was created by subtracting the averaged left and right infraorbital electrodes from the averaged Fp1 and Fp2 electrodes.

To isolate the activities of the left and right visual cortices, a left occipital (LO) signal was calculated by averaging the channels O1, PO3, and PO7, and a right occipital (RO) signal was calculated by averaging channels O2, PO4, and PO8. LO and RO were then convolved with a 10-Hz complex Morlet wavelet to obtain time-domain power and phase signals (Cohen, 2014). A peak-to-peak amplitude signal was then calculated by doubling the square root of the power signal.

The Morlet wavelet was constructed with a center frequency of 10 Hz containing 2^2^/_3_ cycles (the recommended minimum; Cohen, 2019). This cycle count was chosen to optimize temporal resolution and resulted in a wavelet with a full-width at half maximum (FWHM) of 101.6 ms in the time domain and 8.87 Hz in the frequency domain. A FWHM of 8.87 Hz indicates that the wavelet convolution was effectively a bandpass filter with half-power points of 5.57 and 14.44 Hz, approximating the α frequency band.

**Figure 3.**
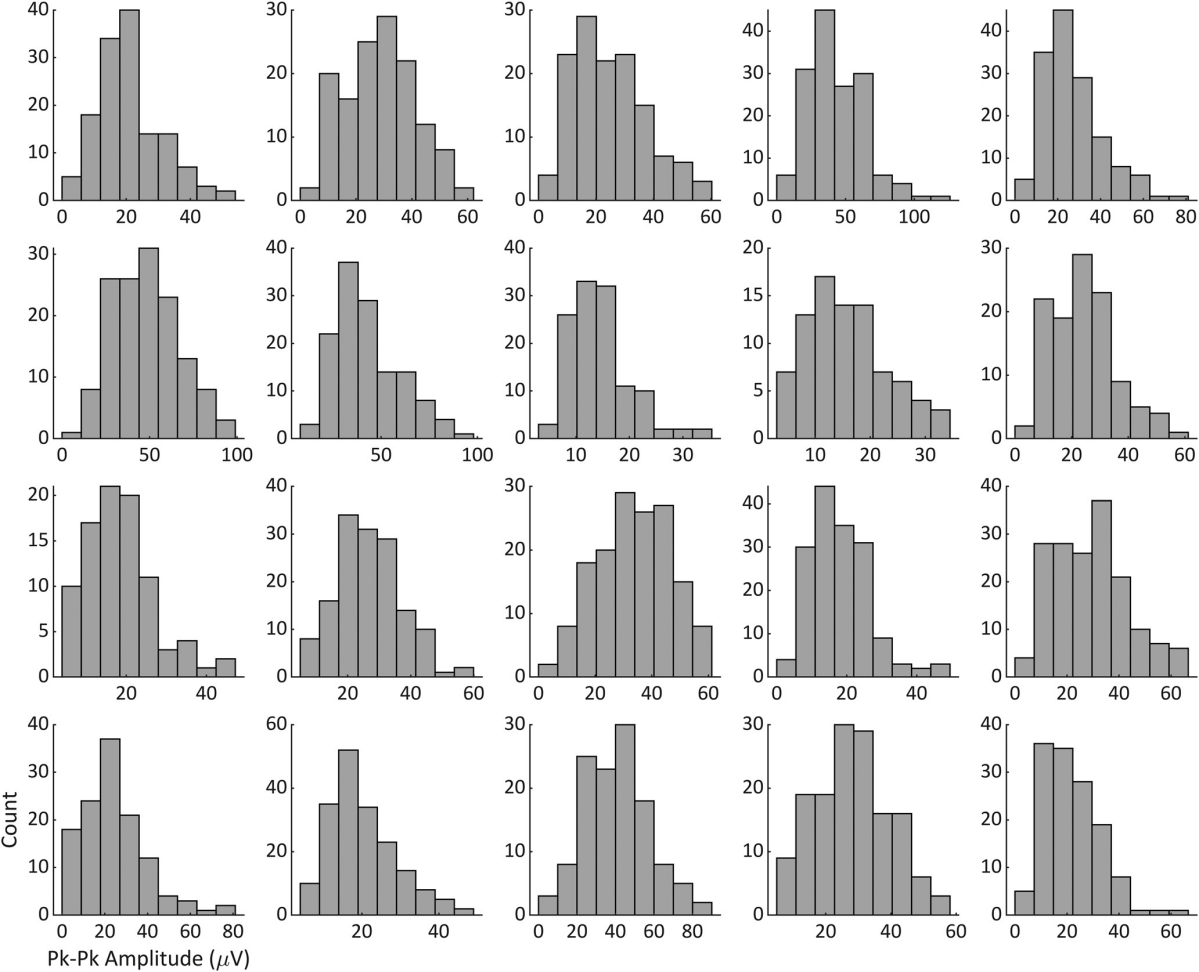
Each of the 20 participants’ α peak-to-peak amplitude distribution for all trials used in the analysis at *t2*.

The following analysis was then performed independently for each participant. Amplitude and phase were measured from LO and RO at the individuals’ *t2* relative to stimulus onset for both observed and missed trials. Trials in which LO and RO phases differed by >90° were rejected from further analysis to control for α asynchrony between the hemispheres. For each of the remaining trials, a single amplitude and phase value was obtained by averaging the amplitude and phase of the LO and RO signals. Phase values were averaged using appropriate statistics for circular quantities (Berens, 2009). These trials were then divided by median amplitude into high-amplitude and low-amplitude bins and then further divided by phase using 90° bins centered on 0°, 90°, 180°, and 270° phase angles. The amplitude median was chosen to provide equal sample sizes in each amplitude bin, and since it is appropriate regardless of distribution normality. When the individual amplitude distributions of our 20 subjects were examined, they appeared to be unimodal and approximately normal with no point of division appearing to be more advantageous than the median (Fig. 3). ORs (the percentage of trials in which the participant observed the stimulus) were then calculated for every bin, and the overall OR across all bins (i.e., the average OR over all trials) was subtracted from these values to obtain a ΔOR value for each of the two (amplitude) × four (phase) bins. Note that ΔOR is not a measure of percent change, but simply a difference of the overall OR and the condition-specific OR.

#### Statistical analysis

This analysis resulted in a within-subjects 2 × 4 repeated measures design with 20 participants and two factors: amplitude (high, low) and phase (0°, 90°, 180°, 270°). These data were analyzed using a repeated measures ANOVA, testing both main effect of amplitude and phase as well as their interaction. Independent *t* test and Tukey’s HSD tests were used to probe any effects found to be significant. All statistical analyses were conducted using MATLAB’s Statistics and Machine Learning Toolbox (R2019b; The MathWorks).

## Results

### Individual retina-to-V1 conduction delay (*t2*) measurement

**Figure 4.**
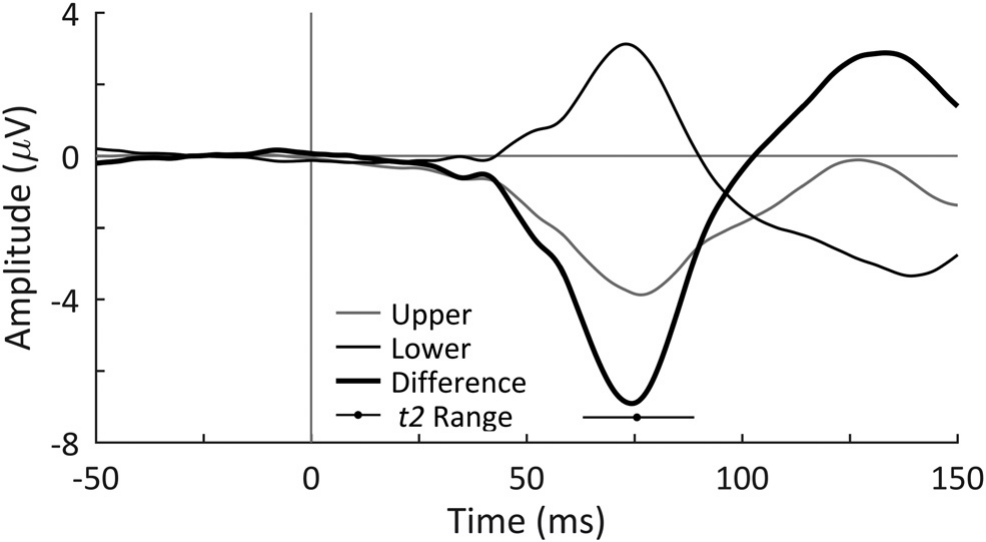
C1 VEP component averaged across all participants in response to upper and lower visual field stimuli. The difference waveform is the lower waveform subtracted from the upper waveform. The mean and range of each participant’s estimated retina-to-V1 conduction delay (*t2*) is indicated and was calculated as the peak latency of the C1 component in each individual’s difference waveform.

In the first part of the experiment, the goal was to estimate each participant’s retina-to-V1 conduction delay, *t2*. To calculate that, we measured in each participant the averaged VEPs in response to upper and lower visual stimuli and calculated the “difference waveform” (based on the difference between two signals); the peak latency of this difference waveform was taken as the participant’s *t2* (Fig. 4). The mean *t2* for all participants was 75.56 ms [range = [62.99, 88.87] ms, 95% confidence interval (CI) = [72.44, 78.69] ms]. These data indicate that it takes, on average, 75.56 ms for visual information to be transmitted from the retina to area V1. For each participant, the phase and amplitude measurements were made at the individual’s *t2* relative to the stimulus onset to assess visual sensitivity.

### OR measurement

The second part of the experiment aimed to investigate whether perceptual performance, when measured at *t2*, is higher at an α wave trough (i.e., phase angle of 270°) than an α wave peak (i.e., phase angle of 90°). To examine this, we presented light flashes to each participant through closed eyes. Light flashes were near individual threshold intensity estimated using the staircase method (Cornsweet, 1962) during a calibration task before the main task to prevent ceiling and floor performance effects. This resulted in the stimuli of average luminance across participants of 6.48 lux. Use of these near-threshold stimuli resulted in total ORs averaging 58.28% across participants.

On average, participants were presented 161.65 stimuli during the main task (range = [120, 200]) with an average interstimulus interval of 12.52 s. LO and RO amplitude and phase signals were measured for each participant and analyzed at the participant’s *t2* time point. Trials in which LO and RO phases differed by >90° were rejected from further analysis resulting in 135.75 trials remaining per participant on average (range = [85, 183]). The amplitude measures were split by their median value into two bins (high and low), and the phase measures were split into four 90° bins centered on 0°, 90°, 180°, and 270° phase angles. A within-subjects two (amplitude) × four (phase) repeated measures ANOVA analysis was conducted on the participant ΔOR values (calculated as the OR per bin minus the overall OR). Mauchly tests indicated no significant violation in the assumption of sphericity for phase (χ^2^(5) = 10.169, *p *=* *0.0706), or the interaction of phase and amplitude (χ^2^(5) = 3.250, *p *=* *0.6615). The ANOVA analysis showed that phase and the interaction between phase and amplitude have statistically significant effects on ORs, as shown in Table 1. Accordingly, these ANOVA results support our hypothesis that a relationship between α phase and visual observation exists, and that this relationship is modulated by the α wave amplitude.

**Table 1:** Two (amplitude) × four (phase) within-subjects repeated measures ANOVA results

Source	Statistic	*p*	$\eta_{p}^{2}$	Power
Phase	*F*_(3,57)_ = 7.971	0.0002	0.2955	0.986
Amplitude	*F*_(1,19)_ = 1.751	0.2014	0.0844	0.242
Phase × amplitude	*F*_(3,57)_ = 3.770	0.0154	0.1656	0.786

**Figure 5.**
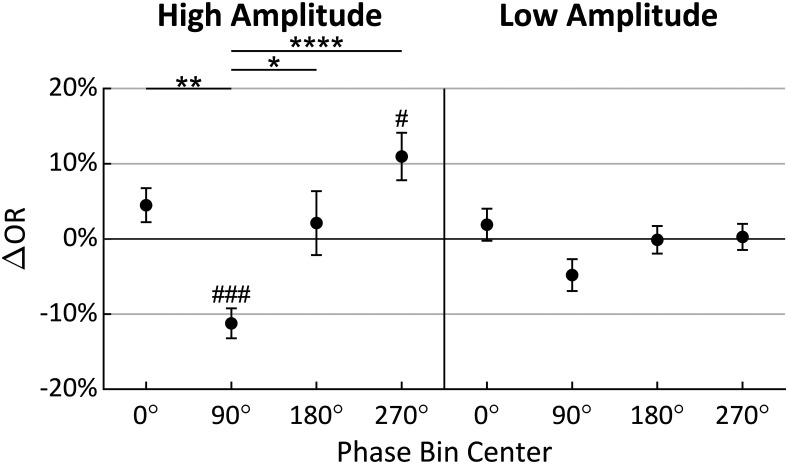
Mean change in OR (ΔOR) for each amplitude and phase condition relative to mean OR across all conditions when analysis was conducted at *t2* (error bars indicate standard error). Tukey’s HSD tests were performed between phase levels within amplitude conditions with significance indicated by *. Bonferroni corrected *t* tests compared each mean to zero with significance indicated by #; 90° = peak; *,#*p *≤* *0.05; **,##*p *≤* *0.01; ***,###*p *≤* *0.001; ****,####*p *≤* *0.0001.

Multiple comparison tests were performed to investigate the precise nature of the effects found to be significant in the ANOVA. Figure 5 shows the results of independent *t* tests (significance indicated by #) comparing ΔOR to zero in each of the phase and amplitude levels. Only phase bins 90° and 270° in the high-amplitude condition were found to be significantly different (*t*_(19)_ = −5.632, *p *=* *0.0002; *t*_(19)_ = 3.466, *p *=* *0.0207, respectively, Bonferroni corrected for eight comparisons; Bland and Altman, 1995). Tukey’s HSD tests (indicated by *) then compared ΔOR distributions between phase bins within each amplitude level. These tests found significant differences in the phase bins only in the high-amplitude condition. Specifically, the 90° phase bin was statistically different from the 0° (*p *=* *0.0077), 180° (*p *=* *0.0308), and 270° (*p *<* *0.0001) phase bins in the high-amplitude condition (Fig. 5). Importantly, the greatest difference in the high-amplitude condition was found between phase bins 90° and 270°, with a 22.20% increase in ORs (95% CI = [9.77%, 34.63%]; Fig. 5). Collectively, these results show that stimuli are observed with greatest probability when the α wave is near a trough at *t2*, and with lowest probably when near a peak, when α amplitude is high. The perception-phase relationship is robust only at high α wave amplitude, likely because during low-amplitude oscillations the measured phase is less representative of the underlying neural population activity.

### Phase-varying analysis of visual observation

**Figure 6.**
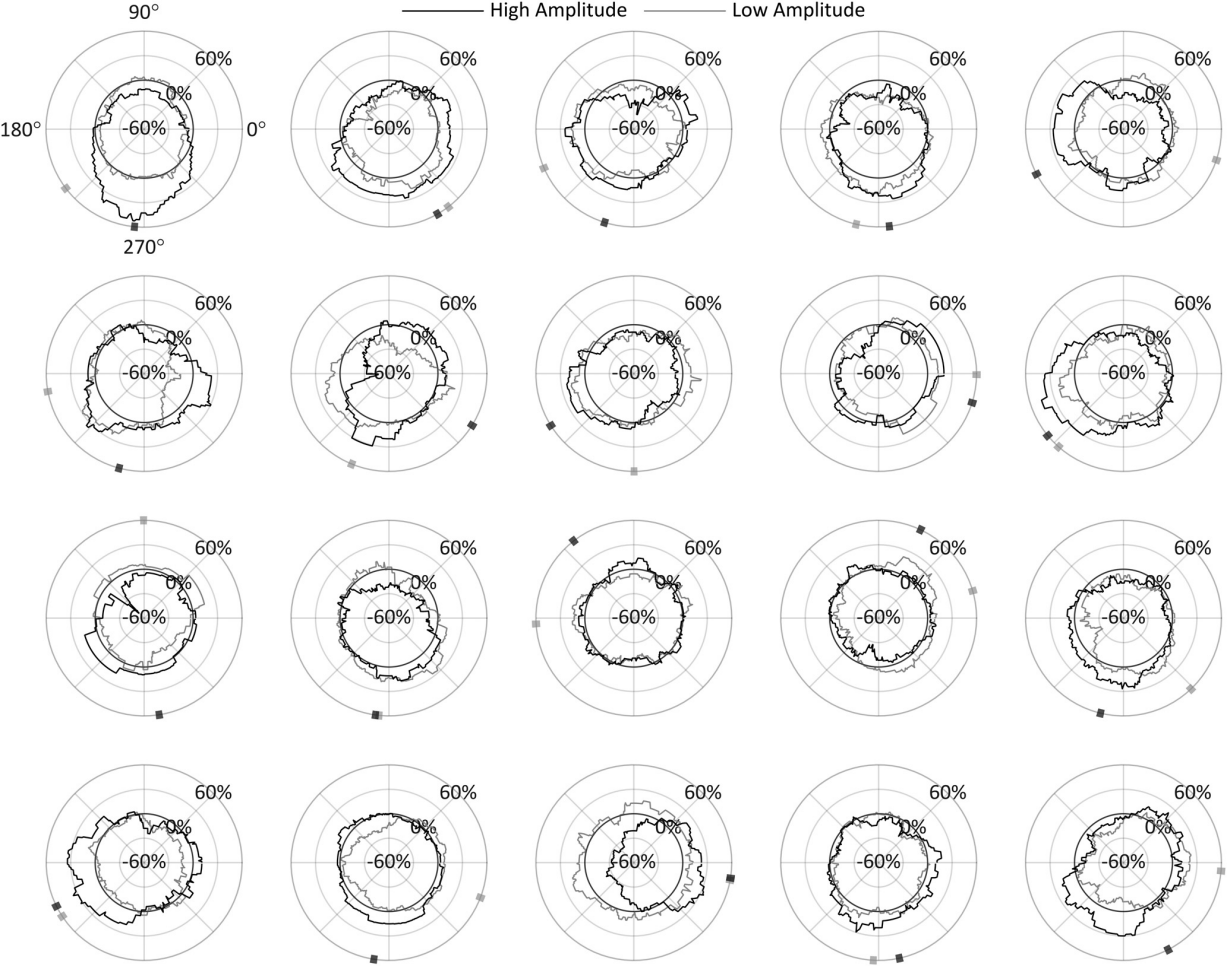
ΔOR as a function of phase for each participant in the high-amplitude and low-amplitude conditions with radial axis extending to negative values for analysis conducted at *t2*. The preferred phase is indicated on the circumference, calculated as the direction toward the circle’s center of mass; 90° = peak.

**Figure 7.**
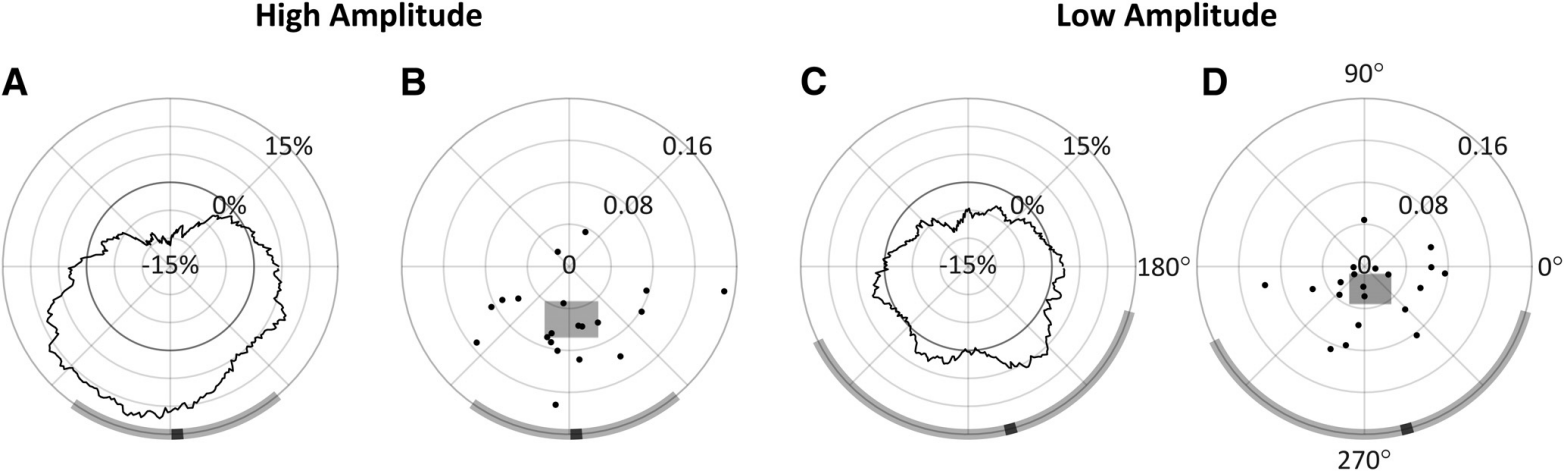
Analysis conducted at *t2.* ***A***, ***C***, ΔOR as a function of phase, with radial axis extending to negative values. ***B***, ***D*,** Individual participants’ preferred phase angles; their magnitude is proportional their individual PPE. The width and height of the shaded rectangles indicate, respectively, the *x* and *y* 95% CI in cartesian space. In all panels, the group mean preferred phase is indicated in black on the circumference of the diagrams, with the shaded region indicating estimated 95% CIs for each amplitude condition; 90° = peak.

For a high-resolution characterization of the phase effect on ΔOR, we repeated the original procedure at *t2* using 90° phase bins rotated in 1° increments for all 360° phase angles. Figure 6 shows ΔOR for each of the 20 participants in both the high-amplitude and low-amplitude conditions as a function of the centered α phase angle of the bins. Indicated along the circumference is the direction of the center of mass of these phase diagrams for each condition, calculated as the angle of the vector mean of each of the 360 points forming the ΔOR distribution. This angle is referred to here as the preferred phase and indicates the phase at which stimuli have the highest probability of being observed given the whole distribution. Note that in the high-amplitude condition, the preferred phase is between 180° and 360° (the negative-amplitude portion of the α cycle) in 18/20 participants. These preferred phase angles have associated magnitudes indicating how far the distribution’s center of mass is offset in that direction and the strength of the effect of phase on ORs. These preferred phase angles and magnitudes for all participants are shown in Figure 7*B*,*D*.

Figure 7*A*,*C* shows the ΔOR distributions averaged across all participants. Indicated in black along the circumference is the group’s preferred phase. Note that calculating the group’s preferred phase as the direction to the center of mass of the group’s ΔOR distributions (Fig. 7*A*,*C*) is equivalent to calculating the vector mean of the 20 individual preferred phases and associated magnitudes (Fig. 7*B*,*D*). By taking the ΔOR value at the preferred phase angle and subtracting from it the corresponding opposite phase angle (by 180°, referred to as the pessimal phase), we calculated the preferred phase effect (PPE), which indicates the increase in OR from pessimal to preferred α phase angles, relative to *t2*. Our results showed that the preferred phase was similar in both the high-amplitude and low-amplitude conditions (272.41° and 284.65°, respectively) with a PPE of 20.96% in the high-amplitude condition but 9.12% in the low-amplitude condition. These data further substantiate an interaction effect on observation probability between α wave phase and amplitude, which was observed in the prior ANOVA analysis.

Additionally, we estimated the 95% CIs for the group’s preferred phase, which are shown as the shaded regions along the circumference of the phase diagrams in Figure 7. These CIs could only be estimated since there is not a known method of computing an angular CI on non-unit vectors directly. To estimate these CIs, the 20 individual preferred phase vectors (with associated magnitudes) were first converted to cartesian values. Then, two 95% CIs were calculated separately on both the *x* and *y* components, indicated, respectively, as the width and height of the shaded rectangles in Figure 7*B*,*D*. The angular interval that encompassed both CIs was then used to represent the estimated 95% CI on the preferred phase in the polar space, although this interval will not necessarily be centered on the preferred phase (i.e., mean). In the high-amplitude condition, this resulted in an estimated 95% CI of [234.75°, 309.84°], and in the low-amplitude condition a much wider CI of [206.22°, 344.45°]. Together with Figure 7*A*,*B*, these results show that observations rates are highest at the trough of the α wave, and lowest at the peak. This effect is prominent in the high-amplitude condition, but also to a much lesser extent in the low-amplitude condition recalling these effects did not reach significance in the prior ANOVA.

### Measuring induced activity

**Figure 8.**
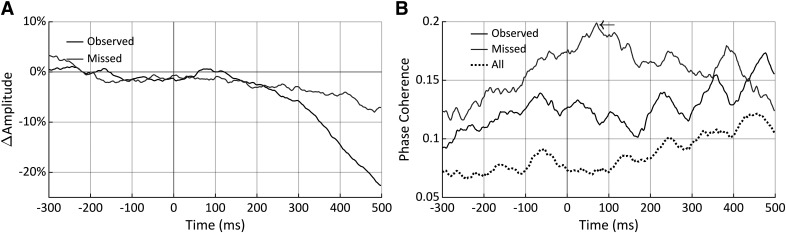
Change in α amplitude, relative to a −300 to −100 ms baseline interval (***A***), and α phase coherence for observed, missed, and all stimuli together (***B***). The arrow indicates a peak in the missed trial phase coherence reflecting the time point were α phase best predicts that a trial will be unobserved by the participant. There was no indication of stimulus-induced effects at the *t2* time point where phase and amplitude measures were hypothesized to be of mechanistic relevance.

α Band desynchronization (ERD) and phase resetting are characteristically observed in response to relevant visual stimuli (Klimesch et al., 2011). These reflect neural processes resulting from the stimulus, not spontaneous activity, and so in our case are only expected in response to observed trials, not missed trials. To ensure that the amplitude and phase measurements made here are the result of spontaneous brain activity and not caused by the stimulus itself, we verified that a drop in amplitude or sudden change in phase coherence across trials does not occur in the observed trials at *t2*. Thus, we first calculated the percent change in α amplitude, relative to a prestimulus baseline period of −300 to −100 ms, for both the observed and missed trials and these are shown in Figure 8*A*. There was the expected characteristic drop in α amplitude (i.e., ERD) in response to the observed stimuli; however, the this was not observed until after 100 ms poststimulus, which did not drop below that of the missed stimuli until after 200 ms. This result indicates that measurements made at *t2* (whose mean value is 75.56 ms and range = [62.99, 88.87] ms) were safe from stimulus induced amplitude effects.

To ensure that there was no event related phase resetting, we next calculated phase coherence across all trials, as well as just the observed and missed trials (Fig. 8*B*). As shown in Figure 8*B*, no phase resetting was apparent in the observed trials or in all trials together, especially before 150 ms. No event related phase resetting would be expected in the missed trials, and this was not seen. However, in the phase coherence across missed trials, there is a prominent peak measured at the 70.31-ms time point, marked by the arrow in Figure 8*B*, but this not an event related phase reset as will be described further in Discussion. In sum, Figure 8 confirmed that no event related amplitude change or phase reset occurred before 100 ms poststimulus, demonstrating that the phase and amplitude measurements taken in the present study are not the result of stimulus induced activity but of ongoing spontaneous oscillations.

### *t2* measurements versus prestimulus measurements

To evaluate the benefit of using the individual *t2* time point for measuring the α phase effect on perceptual performance, we repeated the above analysis at a time point 100 ms before stimulus onset (*t* = –100 ms) and compared the results to those obtained above at *t2*. These measurements have been taken in previous studies in prestimulus periods or at stimulus onset to avoid measuring induced oscillatory effects as mentioned above (Mathewson et al., 2009, 2012; Milton and Pleydell-Pearce, 2016; Kizuk and Mathewson, 2017). Whereas in the previous analysis, the four phase bins centered at 0°, 90°, 180°, and 270° were chosen a priori based on their theoretical significance at the *t2* time point, no prediction could be made about the relevant phase bins at this prestimulus time point. Instead, the analysis was first performed in 90° phase bins in 1° increments, as was performed previously (i.e., in Fig. 7), and the four relevant phase bins were chosen empirically based on the observed preferred phase in the high-amplitude condition. In other words, the phase bins were chosen *post hoc* to reveal the highest effect phase has on amplitude for this prestimulus *t* = –100 ms time point.

**Figure 9.**
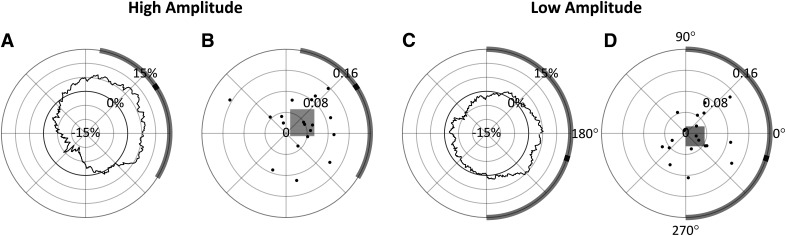
Analysis conducted at *t* = −100 ms. ***A***, ***C***, ΔOR as a function of phase, with radial axis extending to negative values. ***B***, ***D***, Individual participants’ preferred phase angles; their magnitude is proportional their individual PPE. The width and height of the shaded rectangles indicate, respectively, the *x* and *y* 95% CI in cartesian space. In all panels, the group mean preferred phase is indicated in black on the circumference of the diagrams, with the shaded region indicating estimated 95% CIs for each amplitude condition; 90° = peak.

The results of this analysis performed over all 90° bins in both the high-amplitude and low-amplitude conditions are shown in Figure 9. In the high-amplitude condition, a phase effect on perceptual performance can again be observed, however in this case the preferred phase was measured at 33.53° (95% CI = [−31.69°, 80.29°]) with a 15.48% PPE. In the low-amplitude condition, the preferred phase was measured at −17.22° (95% CI = [−90.04°, 90.07°]) with a 5.22% PPE. Based on the preferred phase measured in the high-amplitude condition for prestimulus *t* = –100 ms, the four 90° phase bins centered at 33°, 123°, 213°, and 303° were therefore chosen for the 2 × 4 repeated measures ANOVA analysis. Mauchly tests indicated no significant violation in the assumption of sphericity for phase (χ^2^(5) = 5.194, *p *= 0.3926), or the interaction of phase and amplitude (χ^2^(5) = 8.861, *p *=* *0.1147). As shown in Table 2, only the main effect of phase was found to have a statistically significant effect on ORs. Despite these phase bins showing the highest phase effects at *t* = –100 ms, phase, whose bins were chosen a priori based on theoretical significance, had a stronger effect on observation when the measurements were taken at *t2* (with roughly twice the effect size).

**Table 2:** Two (amplitude) × four (phase) within-subjects repeated measures ANOVA results repeated at 100 ms before stimulus onset

Source	Statistic	*p*	$\eta_{p}^{2}$	Power
Phase	*F*_(3,57)_ = 3.378	0.0243	0.1510	0.735
Amplitude	*F*_(1,19)_ = 0.008	0.9289	0.0004	0.051
Phase × amplitude	*F*_(3,57)_ = 1.695	0.1784	0.0819	0.420

**Figure 10.**
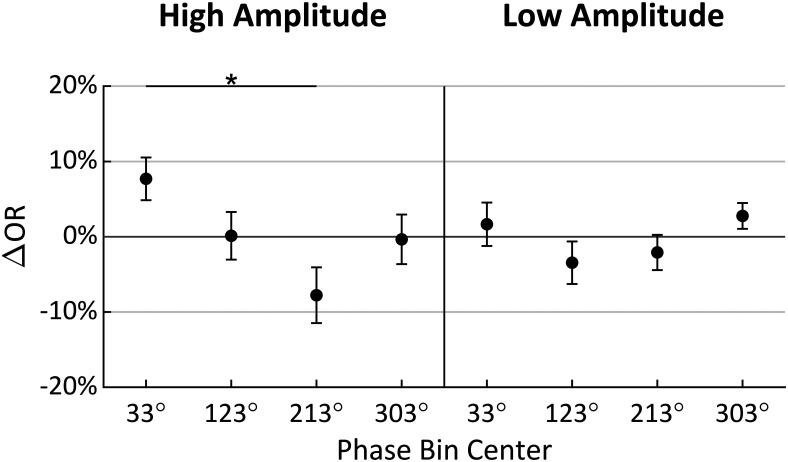
Mean change in OR (ΔOR) for each amplitude and phase condition relative to mean OR across all conditions when analysis was conducted at *t* = −100 ms (error bars indicate standard error). Tukey’s HSD tests were performed between phase levels within amplitude conditions with significance indicated by *. Bonferroni corrected did not find any of the means to significantly differ from zero; 90° = peak; *p ≤* *0.05.

Figure 10 shows the multiple comparisons result for this data collected at 100 ms before stimulus onset for better comparison to those from *t2* shown in Figure 5. Bonferroni corrected independent *t* tests compared each ΔOR value to zero, but none were found to be significant, indicating that none of the ORs in any of these four phase bins differed from the overall mean OR either amplitude condition. Tukey’s HSD tests (indicated by *) were used to compare ΔOR distributions between phase bins within each amplitude level, but these tests only found a significant difference between 33° and 213° (*p *=* *0.0143), where there was a 15.47% difference in observations rates (95% CI = [2.39%, 28.55%]).

In our original hypothesis, we predicted that the high-amplitude preferred phase would be 270° at *t2*, which was approximately *t* = 75 ms on average. Assuming (1) a perfectly stationary and (2) an exactly 10-Hz signal, we would have predicted that the preferred phase at *t* = –100 ms, as well as at *t0*, would have been 0°. This was approximately what was found here; however, the inexactness is expected since neither assumption is exactly met in the natural α rhythm. The non-stationarity would additionally explain the decrease in effect size from *t2* to *t* = –100 ms. Collectively, these results show that although an α phase effect on ORs in a prestimulus period can be observed, the strength of the effect is much weaker than when measurements are taken at *t2*. This finding shows that measurement of α oscillation amplitude and phase have a stronger correlation with visual perception at *t2* than at *t* = −100 ms.

## Discussion

This study presents a novel, controlled method for examining the neuronic shutter effect, hypothesized to be reflected in the posterior α oscillations observed in human EEG. Our hypothesis was that perceptual performance is modulated by the α rhythm in a precise phase and timing relationship. To test this relationship, we (1) presented near-threshold intensity visual stimuli through closed eyes to participants; (2) accounted for individual retina-to-V1 conduction delays (*t2*); (3) controlled for asynchrony between hemispheres; and (4) measured the α phase and amplitude relative to *t2*. Our results confirm the hypothesized relationship between the α rhythm phase and perception, with the greatest rates of observation occurring at the α wave trough (excitatory state) and the lowest rates at the α wave peak (inhibitory state). The perception-phase relationship appears to be modulated by α amplitude as it is observed at high, but not low, α wave amplitude. This work is novel in considering the underlying neural structure and function of the visual system when predicting the exact phase relationship to perception, and in controlling for individual neural conduction delay and asynchrony between hemispheres.

While the α wave shutter effect has been previously examined (Dustman and Beck, 1965; Mathewson et al., 2009, 2012; Milton and Pleydell-Pearce, 2016; Kizuk and Mathewson, 2017), this is the first study, to our knowledge, to directly examine the relationship between perceptual performance and spontaneous α phase as measured relative to each individual’s conduction delay. Previous studies have either measured α phase at stimulus onset, *t0*, or during prestimulus periods (Mathewson et al., 2009, 2012; Milton and Pleydell-Pearce, 2016; Kizuk and Mathewson, 2017). Accordingly, the phase measurements were not always well aligned with the LGN excitability state according to the proposed cellular mechanism. Here, when we accounted for each individual’s conduction delay, as opposed to the group average conduction delay or measuring at stimulus onset *t0*, we demonstrated the existence of a hypothesized relationship between the α phase and perception. Notably, these results provide the most robust evidence consistent with cyclic LGN excitability as the cellular mechanism mediating this effect, although we do not directly examine observe this mechanism. Dustman and Beck (1965) measured reaction times, as opposed to ORs, at α phase adjusted to group average, as opposed to individual conduction delay. This study, similar to ours, found slowest reaction times at the peak (90°) of the α wave, and fastest reaction times close to the α trough (240°).

A 2 × 4 repeated measures ANOVA found that α phase at *t2* had a significant effect on perceptual performance as hypothesized. The interaction effect of phase and amplitude was also found to be significant; the multiple comparisons tests in Figure 5 show that low amplitude attenuates the phase effect. This supports the hypothesis that low-amplitude α waves reflect asynchronous inhibition within the visual system. Although not part of our original hypothesis, it is somewhat surprising that no main effect of amplitude was found since increased α power has sometimes been found to predict poorer perceptual performance (Ergenoglu et al., 2004; Mathewson et al., 2009; Brüers and VanRullen, 2018). This perception-power relationship is usually attributed to more generalized early visual inhibition as evidenced by the attenuation of early (C1 and N150) VEP components with increased α power (Iemi et al., 2019). Figure 7 gives a higher phase angle resolution view of the effects seen in Figure 5. In both the high-amplitude and low-amplitude conditions, the preferred phase was near the wave trough (272.41° and 284.65° at high amplitude and low amplitude, respectively). However, in the low-amplitude condition, the 95% CI of the preferred phase was much wider, and the PPE was lower (20.96% and 9.12% at high and low amplitude, respectively). These results show how α amplitude modulates the effect of α phase on perception.

To demonstrate the significance of taking α amplitude and phase measurements at *t2*, we repeated our analysis at *t* = −100 ms and compared them to those obtained at *t2*. This prestimilus time point was chosen for two reasons. First, given the risk in poststimulus measurements getting altered by stimulus processing, a prestimilus time point is, therefore, “safer” than poststimulus or stimulus-onset time points. Second, given that α oscillations have a primary frequency of 10 Hz (i.e., has a period of 100 ms), the amplitude-phase relationship would repeat roughly every 100 ms. Thus, our results at *t* = –100 ms could help to predict this relationship at stimulus onset, which is 100 ms (or one period) away but under safer signal analysis conditions. At prestimulus time point of −100 ms (*t* = −100 ms), we did find a significant phase effect, but by using different phase bins more relevant to that time point (i.e., 33°, 123°, 213°, 360°). These phase bins were chosen *post hoc* to reveal the highest effect phase has on observation for this prestimulus time point. Despite showing the highest phase effects, a phase whose bins were chosen a priori based on theoretical significance had stronger effects on observation when the measurements were taken at *t2*. Specifically, the new analysis shows that while phase was found to have a statistically significant effect on ORs between the preferred and pessimal phase bins in the high-amplitude condition, the effect size was weaker and roughly half of that at *t2*. These results show the advantage of making phase and amplitude measurements at *t2* rather than in prestimulus periods.

Poststimulus measures of ongoing spontaneous oscillations are generally avoided since there is a risk that they will be altered by stimulus processing. We verified that our *t2* amplitude and phase measurements were not influenced by that by measuring α band ERD and phase coherence over the −300- to 500-ms time range relative to stimulus onset (Fig. 8). No stimulus related ERD or phase coherence were observed before 100 ms indicating that our measures at *t2* were independent of stimulus processing responses and instead reflected spontaneous activity. Interestingly, there was an overall increase in phase coherence values of the observed and missed trials compared with that of all trials combined (Fig. 8*B*). This increase is expected since, according to the phase effects on ORs found above, trials that are observed and missed will predominantly occupy specific, opposing phase ranges; thereby resulting in relatively high phase coherence across trials when grouped by observational outcome, and low phase coherence when grouped together. It is also important to note that the missed trial phase coherence trace features a prominent peak at 70.41 ms (Fig. 8*B*, arrow). We do not interpret this peak as an event related phase reset since (1) it is the peak of an upward trend beginning at least 300 ms before stimulus onset, (2) a decreasing trend is observed immediately afterward, and (3) no phase reset would be expected in response to an unobserved stimulus. Instead, we interpret this peak as marking the time where phase most accurately predicts that a stimulus will not be observed, which, in support of our hypothesis, is within 5 ms of the average estimated *t2* (75.56 ms), the point hypothesized to best predict observational outcome.

### Neural mechanism

**Figure 11.**
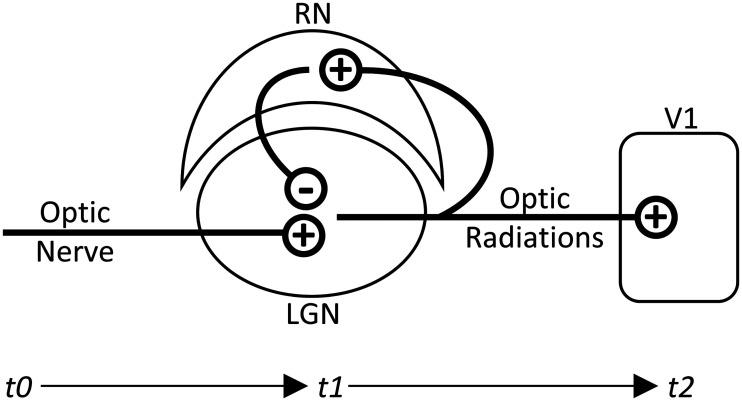
Visual pathway from retina to V1. The LGN/RN negative feedback loop provides a mechanism of cycling LGN excitability state. This LGN/RN network is known to give rise to sleep spindles. The same, or a similar, mechanism is expected to give rise the posterior α rhythm.

Understanding the biological mechanism underlying α oscillation is important for explaining its function. Although simultaneous recordings with fMRI and PET correlated α activity with fluctuations in the thalamus, thus suggesting this might be the origin of α rhythm (Hughes et al., 2004; Omata et al., 2013), so far the precise mechanism in humans is not yet known. However, sleep spindles, a ∼10 Hz α-like oscillation that occurs in 1- to 3-s bursts during transition to sleep, do have a well-established mechanism. The sleep spindle mechanism includes a negative feedback loop between the LGN and the reticular nucleus (RN; Fig. 11). Both nuclei have T-type Ca^+^ channels that, when activated, exhibit burst firing. Between each burst of the LGN, the Ca^+^ channels of the LGN are known to enter a refractory period, and the RN sends a similar, but inhibitory, burst to the LGN. During this time, sensory information from the optic nerve would be less likely to be relayed to V1. Importantly, the refractory periods of these T-type Ca^+^ channels are known to result in each nucleus firing at a ∼10-Hz rate (Lopes da Silva, 1991; Sherman, 2001; Timofeev and Bazhenov, 2005; Alexander et al., 2006; Timofeev and Chauvette, 2011). Each LGN burst firing generates massive EPSPs in V1, measured as a negativity in the occipital EEG (Timofeev and Bazhenov, 2005) and producing the ∼10-Hz oscillation of sleep spindles seen on EEG. Chen et al. (2016) showed that, in mice, very similar mechanisms give rise to both sleep spindles and waking α oscillations and that the mechanism is capable of phasically modulating sensory transfer through the thalamus, and on this basis, they predicted the perceptual results found here. In cats, this mechanism was also found to produce waking α oscillations with the same phasic sensory gating effect (Lorincz et al., 2009). Although additional and alternate sources, including extrastriate cortical sources, have been proposed to give rise to this oscillatory activity (Steriade et al., 1990; Connors and Amitai, 1997; Bollimunta et al., 2008, 2011; van Dijk et al., 2008), there is much evidence supporting this thalamic mechanism as the primary driver (Hughes and Crunelli, 2005). Although the present study does not directly test this thalamic mechanism, it is offered as a purely speculative explanation for our results on the basis of previous and more direct research findings (Lorincz et al., 2009; Chen et al., 2016).

### Cellular shutter effect generates a behavioral graded gating effect

Although our speculation and not directly tested here, if the neural mechanism posited above were correct, the neuronic shutter effect would then best be described as occurring at the level of individual LGN relay cells, in which the effect may occur at a nearly binary level. However, the behavioral effects we observe are graded, with increasing and decreasing probabilities of stimulus observation. This may be explained given that oscillating excitatory states are not always synchronized across all LGN cells. It is not necessarily the case that all cells are simultaneously in burst-mode firing state; some may concurrently operate in the normal tonic firing state. Thus, one cell may be in a state of inhibition and not relay to V1, while others may relay visual information as usual. However, in asynchronous states, the α rhythm should be decreased in amplitude. As shown in this study, at higher α amplitudes, the differences in ORs between peak and trough phases at *t2* increased, presumably indicating that the measured phase becomes more representative of the excitatory state of larger populations of LGN cells with increasing α amplitude. Asynchrony is also important to consider, not just among cells within an LGN but between the LGNs of the left and right hemispheres. As on a cellular level, one LGN may restrict the flow of visual information while the other relays it. Presumably, if all cells within and between LGNs were in perfect synchrony, behavior relative to peak/trough phase at *t2* would reflect a perfect on/off visual shutter. This simple relationship was not observed in this study, and it is therefore more appropriate to describe the proposed neuronic shutter effect at the cellular level generating a graded, gating effect of perception at the behavioral level.

In sum, to our knowledge, this is the first study to examine the neuronic shutter effect with an exact phase and timing relationship to perceptual performance predicted by the underlying physiology of the visual system. The study design is novel and rigorous in controlling for individual visual conduction delays and hemispheric asynchrony. During times of high-amplitude α oscillations, we found participants on average are most likely to perceive stimuli at waveform trough (272.41°) with ORs 20.96% greater than at the opposing peak phase (92.41°). Given the rigor in our phase measurements, these results provide strong support for a mechanism that modulates perception described by the neuronic shutter effect and is reflected in the posterior α rhythm. Further studies with conduction delay-adjusted phase measurement are needed to investigate this shutter effect in the μ and tau rhythms over the sensorimotor and auditory cortices, to explore the possibility of this mechanism in other sensory systems.

## Synthesis

Reviewing Editor: Ifat Levy, Yale University School of Medicine Decisions are customarily a result of the Reviewing Editor and the peer reviewers coming together and discussing their recommendations until a consensus is reached. When revisions are invited, a fact-based synthesis statement explaining their decision and outlining what is needed to prepare a revision will be listed below.

This manuscript examines the influence of amplitude and phase of spontaneous oscillations in the alpha-band on visual detection performance. It tests the intriguing hypothesis that the alpha oscillations have a role in gating the sensory input, and thus that the alpha phase should predict visual sensitivity. Evidence supporting this hypothesis will inform and constrain functional models that posit a role for alpha in attentional gating. Two important aspects distinguish this paper from previous studies on the topic. First, the experiment was conducted while subjects had their eyes closed - a condition under which the alpha rhythm is particularly strong and easier to quantify. Second, the effect of alpha on detection was specifically quantified for the time point at which stimulus information supposedly arrives in V1, at the individual subject level. This is an interesting and well-written manuscript, the methods are generally sound, and the results confirm and extend those of previous studies. The manuscript addresses a timely research topic and its novel procedures are likely to stimulate research in this field. The reviewers had, however, some concerns about the analysis and interpretation of the results which should be addressed, as detailed below.

Major comments

-Data analysis and presentation

1. The authors only present the statistical group results, with no presentation of the collected data before processing, or of single subject statistics. This makes it difficult to assess the strength of the results.

2. The ‘visual sensitivity task’ should be illustrated in the manuscript, together with single trial VEP examples. While the authors’ statement in the methods that the stimulus intensity was at threshold is reasonable, showing some data that support it would be helpful.

3. The median split of the alpha oscillation power is arbitrary. It would be good to show the distribution of alpha power for each subject and see whether there are modes that can be identified and used for posthoc analysis, rather than a median split.

4. Figure 4 needs to be plotted for each subject individually.

-Involvement of LGN/thalamus:

The paper makes the strong claim that alpha oscillations recorded at the scalp level are driven by pacemakers in the LGN and influence perception by interacting with stimulus input in V1. This claim is reflected, for instance, in this statement from the Discussion: “This hypothesis was developed on the basis that cycling LGN excitability is theorized to underlie this oscillation. To test this relationship, we (1) presented threshold intensity visual stimuli ...”. While these claims appear plausible based on previous literature, it is still important to acknowledge that the involvement of the LGN is just a conjecture. The current study did *not* test the relationship between LGN excitability and detection. Doing so would require a direct measure of LGN activity. This needs to be addressed appropriately.

First, thalamic pacemakers are presented as the exclusive generators of cortical alpha oscillations. This is not the case - intrinsic cortical generators (i.e. generators not primariliy driven by thalamic sources) also contribute to the alpha rhythm, see e.g.

Steriade, M., Gloor, P. L. R. R., Llinas, R. R., Da Silva, F. L., & Mesulam, M. M. (1990). Basic mechanisms of cerebral rhythmic activities. Electroencephalography and clinical neurophysiology, 76(6), 481-508.

Connors, B. W., & Amitai, Y. (1997). Making waves in the neocortex. Neuron, 18(3), 347-349.

Bollimunta, A., Mo, J., Schroeder, C. E., & Ding, M. (2011). Neuronal mechanisms and attentional modulation of corticothalamic alpha oscillations. Journal of Neuroscience, 31(13), 4935-4943.

Bollimunta, A., Chen, Y., Schroeder, C. E., & Ding, M. (2008). Neuronal mechanisms of cortical alpha oscillations in awake-behaving macaques. Journal of Neuroscience, 28(40), 9976-9988.

This literature should be acknowledged in the paper. Moreover, given that the drivers of the scalp-recorded alpha rhythm in the present study are unknown, it is impossible to draw strong conclusions regarding the effect of thalamic excitability on detection performance. The empirical findings of this study could have been obtained, in principle, even if the drivers of the recorded alpha rhythm were entirely intrinsic to the cortex. Thus, any conclusions about thalamic involvement (e.g. relating alpha trough to “excited thalamus” and peak to “inhibited thalamus” or the conclusion that “This supports the hypothesis that low amplitude alpha waves reflect asynchronous inhibition within LGN cells.”) should be indicated clearly as speculative and generally be toned down.

Second, the conclusions are based on the assumption that alpha amplitude/phase were generated by a source in primary visual cortex. While this is not entirely implausible given the short latency of the effect, the source of the alpha signal was not actually localized. The mean C1 peak latency was at 75 ms, and it was longer in some subjects. However, visual signals reach visual areas beyond V1 quite rapidly (Schmolesky, M. T., Wang, Y., Hanes, D. P., Thompson, K. G., Leutgeb, S., Schall, J. D., & Leventhal, A. G. (1998). Signal timing across the macaque visual system. Journal of neurophysiology, 79(6), 3272-3278.). Also, higher level visual areas have been reported as sources of the alpha rhythm in general, and of its effect on detection specifically (Van Dijk, H., Schoffelen, J. M., Oostenveld, R., & Jensen, O. (2008). Prestimulus oscillatory activity in the alpha band predicts visual discrimination ability. Journal of Neuroscience, 28(8), 1816-1823.). Thus, V1 as the source of the alpha rhythm is another conjecture and should be indicated as such.

-Interpretation of seeing with closed eyes

Visual stimuli were presented through closed eye-lids. While it is a bit odd to test how well people can see while having their eye closed, the great advantage of this procedure is that the alpha rhythm is a lot stronger than with eyes open. However, the interpretation of the results seems to extend to visual perception with eyes open. This is reflected, for instance, in the following statement: “Given the alpha rhythm’s known negative correlation with attention, these results are consistent with the theory that the alpha rhythm facilitates attention by effectively down-sampling incoming sensory information.”. First, please clarify why and how temporal downsampling would help attention. Second, why is the mechanism supposedly responsible for this downsampling of visual input so much stronger in a situation when visual input does not occur and there is nothing to see? Finally, it will be helpful to mention that future work should compare findings in the same subjects with eyes closed and eyes open.

-How relevant is the specific time point of C1 peak latency?

The effect of oscillatory amplitude and phase was assessed exclusively at the latency of the C1 component of the event-related potential, i.e. at the time the first feedforward volley from the LGN arrives at V1 (referred to as _t2_ in the manuscript). This is, in fact, one of the unique novel aspects of this study. The relevance of _t2_ for the interpretation of the results is reflected in such statements as: “This work is novel in considering the underlying neural structure and function of the visual system when predicting the exact phase relationship to visual sensitivity, and in controlling for individual neural conduction delay ...”. However, limiting the analysis to this single time point can result in potentially misleading conclusions.

First, since the relationship between amplitude/phase and detection is analyzed only at this one time point, it is impossible to assess if there is actually anything specific about _t2_. It is possible that alpha amplitude/phase is correlated with detection just as strongly or more strongly at other latencies. Does knowing each subject’s conduction delay/C1 latency really add any useful information? Given that these questions concern one of your main conclusions, it would be important to substantiate this conclusion by conducting the same analysis for a longer time-interval, both time-locked to _t2_ and to stimulus onset (which most studies in this field have done).

Moreover, your measure of _t2_ is the individual C1 *peak* latency. To assess the optimal phase at the time visual information arrives in V1, would it not be more relevant to quantify C1 *onset*? While the peak is a more robust measure, this pragmatic advantage is offset by the theoretical disadvantage that it is not a good measure of _t2_.

Second, there is a vast literature on the relationship between detection performance and *pre-stimulus* alpha amplitude and phase. Of course, these studies were conducted under the assumption that amplitude and phase of ongoing oscillations only really matter at the time a stimulus is processed in visual cortex, not hundreds of milliseconds before. However, stimulus processing goes along with stimulus-evoked signals (i.e. the ERP) and non-phase-locked changes in spectral power (e.g. alpha-band desynchronization). These stimulus-related responses, which are often further modulated by stimulus detection, may distort measures of presumably “ongoing” oscillations to an unknown extent. Thus, analyzing the post-stimulus time-range has been considered “unsafe” by most studies. Since time-frequency transforms cause some temporal smear, many studies have even restricted themselves to analyzing only pre-stimulus time-ranges sufficiently long before stimulus onset.

The present study analyzed the relationship between detection performance and *post-stimulus* alpha amplitude and phase. While it is clear why the _t2_ time point was theoretically relevant, it is still important to demonstrate that measures of alpha amplitude/phase are not influenced by post-stimulus signals. To this end, it would be important to see how the alpha-detection relationship evolves over a longer time-range (see also previous point). It would be good to see if the same effect (although maybe weaker) is observed in a “safe” pre-stimulus time-range, where most previous studies have reported similar effects. In addition, the time-course of the amplitude/phase-detection effect must be compared to the time-course of alpha-power (i.e. event-related (de)synchronization) and phase-locking/inter-trial coherence. It is important to show that no such signals are observed at the _t2_ latency in the average across all trials, and no differences between detected and undetected stimuli.

Minor comments

1. “Each burst in LGN relay cells results in a large excitatory postsynaptic potential (EPSP) at V1, measured as a negative deflection in the occipital EEG , in which higher amplitudes indicate greater synchrony among LGN cells 51 (Busch et al., 2009; Mathewson et al., 2009; Mathewson et al., 2012).” These papers are cited out of context; they did not investigate or even mention LGN relay cells and their effect on V1.

2. You refer to the effect of phase on perception as the “shutter effect” and cite some of the classical literature from the 1960ies. It would be appropriate to also cite contemporary literature on the topic, such as the perceptual sampling hypothesis, e.g.

VanRullen, R., & Koch, C. (2003). Is perception discrete or continuous?. Trends in cognitive sciences, 7(5), 207-213.

VanRullen, R., Zoefel, B., & Ilhan, B. (2014). On the cyclic nature of perception in vision versus audition. Philosophical transactions of the Royal Society B: Biological sciences, 369(1641), 20130214.

3. Some essential details are missing in the Methods section:

- Report the luminance of the threshold stimuli in cd/m^2 (mean across subjects)

- How many stimuli were presented in total?

- Inter-stimulus intervals.

- “Trials in which LO and RO phases differed by more than 90 degrees were rejected from further analysis” Report the proportion of rejected trials. How many trials remained for analysis?

- Alpha amplitude and phase were quantified at left and right occipital channels. Were the analyses conducted separately for left and right channels? If so, both should be reported. Or were the data somehow combined from left/right channels? If so, it should be explained how they were integrated.

4. The figure legends for Figure 2 and Figure 3 need to be expanded. The reader needs to be able to evaluate the merit of these plots based on the legends alone. Currently, they are not understandable.

5. The EEG data were recorded with Biosemi’s default CMS/DRL reference, but were later re-referenced to average mastoids. Does the choice of reference influence the phase distribution? If phases were reference-dependent, it would not be valid to interpret phase angles measured on the scalp according to the state of a thalamic pacemaker (e.g. “trough corresponds to excitatory volley from LGN”).

6. “This study presents a novel, controlled method for examining the neuronic shutter effect, reflected in the posterior alpha oscillations observed in human EEG, in relation to the cellular mechanism hypothesized to underlie that rhythm.” This sentence confusing; what does “... in relation to” relate to exactly?

7. Perceptual performance was tested with a detection task in which subjects pressed a button when they detected a stimulus and did not respond otherwise. This task is quite common in this field. It may be misleading to refer to the hit rate in this task as “sensitivity”. According to signal detection theory, sensitivity refers to the ability to discriminate stimulus-presence from stimulus-absence. Quantifying sensitivity thus requires a measure of performance on signal absent trials, i.e. the false alarm rate. It is important to distinguish mere hit rate from sensitivity because several studies have demonstrated that ongoing alpha power affects hit rates by modulating subjects’ response criterion, not their sensitivity:

Iemi, L., Chaumon, M., Crouzet, S. M., & Busch, N. A. (2017). Spontaneous neural oscillations bias perception by modulating baseline excitability. Journal of Neuroscience, 37(4), 807-819.

Limbach, K., & Corballis, P. M. (2016). Prestimulus alpha power influences response criterion in a detection task. Psychophysiology, 53(8), 1154-1164.

Please rephrase accordingly.

8. The analysis did not show a significant main effect of alpha power on detection (i.e. lower hit rates for strong alpha power). Given that numerous studies have reported this effect, this negative finding should be briefly discussed.

9. The final paragraph on the relevance of the present findings for neurological disorders may be too far-fetched. While these studies report various properties of the alpha rhythm under neurological disorders, none of them was actually concerned with the kind phase effect reported in the present study. Thus, there doesn’t seem to be a real connection or relevance of this study for neurology. To be clear, this is not a serious shortcoming; the present findings are interesting enough without this connection. It will be better to remove this paragraph and the corresponding statement in the abstract all together.

10. In the interest of Open Science and replicability, the authors are encouraged to make their data publicly accessible (e.g. on OSF.io).
